# Medullary Carcinoma of the Colon: A Histopathologic Challenge

**DOI:** 10.7759/cureus.15831

**Published:** 2021-06-22

**Authors:** Zainab Fatima, Purva Sharma, Bahaaeldin Youssef, Koyamangalath Krishnan

**Affiliations:** 1 Internal Medicine, East Tennessee State University, Johnson City, USA; 2 Medical Oncology, East Tennessee State University, Johnson City, USA; 3 Pathology, East Tennessee State University, Johnson City, USA

**Keywords:** medullary carcinoma, colon cancer, chemotherapy, metastasis, microsatellite instability, immunohistochemistry

## Abstract

Medullary carcinoma (MC) of the colon is a rare and unique histologic subtype of colorectal cancer. It is commonly associated with deficient mismatch repair proteins and has a strong association with Lynch syndrome. Diagnosis is challenging as it does not have the usual immunohistochemical stains on pathology seen in colorectal adenocarcinoma. Here, we discuss an interesting case of MC of the colon that was metastatic on presentation and constituted a diagnostic challenge.

## Introduction

Medullary carcinoma (MC) of the colon is a rare subtype of colon carcinoma which includes poorly differentiated and undifferentiated histology. The incidence of MC is estimated to be 0.03% of all colorectal carcinomas (CRC) [1]. MC is twice as prevalent in middle-aged women, favors the right side of the colon, and has a lower risk of lymph node metastasis than colon adenocarcinoma. MC can be poorly differentiated (72%) or undifferentiated (22%) with solid sheets of cells and excessive intraepithelial lymphocytic infiltration [2]. It does not have the glandular structure seen in adenocarcinoma [2]. Only a few cases of metastatic MC have been published in the literature [2,3]. We present a rare case of MC, which was metastatic at presentation, and the diagnostic challenge it posed as the tumor cells lacked the usual markers for intestinal differentiation, such as cytokeratin (CK)20 and special AT-rich sequence binding protein 2 (SATB2). Loss of mismatch repair proteins and expression of calretinin helped validate the diagnosis.

## Case presentation

A 77-year-old female with a history of hypertension, hyperlipidemia, and osteopenia presented with a four-month history of progressively worsening lower abdominal pain. Abdominal pain radiated to the back and was associated with vomiting and abdominal distention. The patient also reported excessive fatigue and weight loss of 10 pounds over two months. Physical examination was significant for diffuse abdominal tenderness with rebound tenderness in the right upper quadrant and periumbilical region.

**Figure 1:**
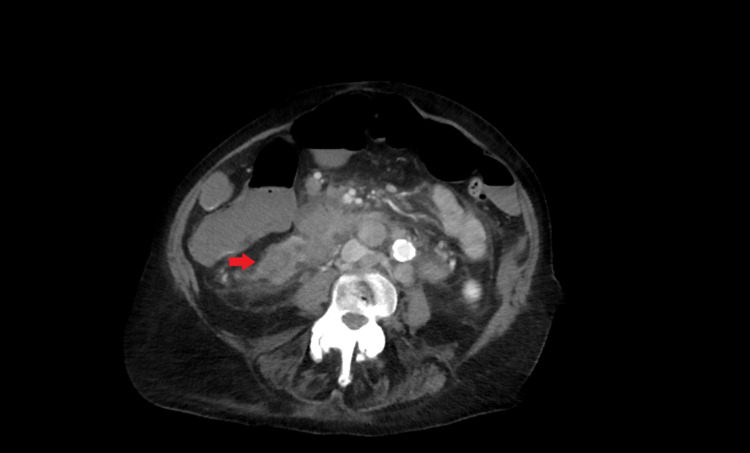
Enlarged right lower quadrant mass measuring 9.1 × 4.0 cm.

**Figure 2:**
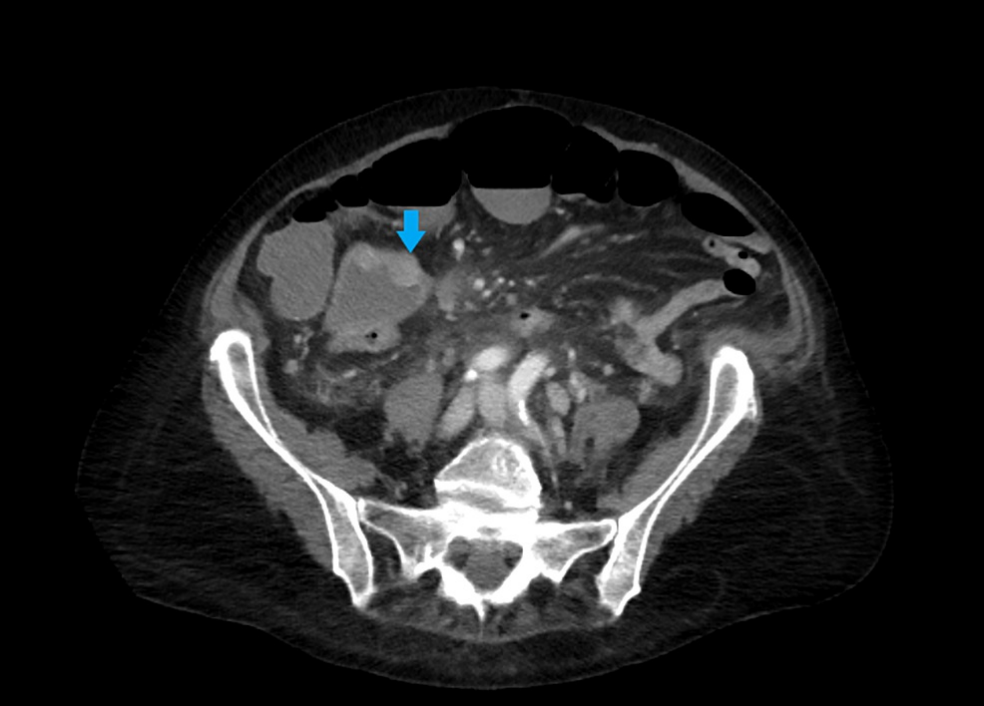
Nodular thickening in the cecum.

Laboratory evaluation was unremarkable, including complete blood count and chemistry panel. Computed tomography (CT) scan of the abdomen/pelvis with contrast showed a 9.1 × 4.0 cm mass in the right lower quadrant, associated with thickening of the anterior wall of the cecum with mesenteric and retroperitoneal nonbulky lymphadenopathy (Figures 1, 2).

**Figure 3:**
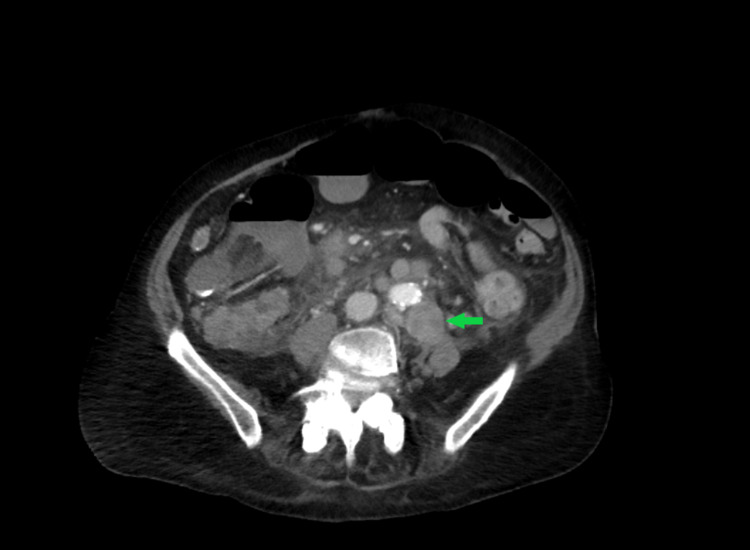
Enlarged para-aortic lymph node measuring 3.1 cm.

CT also showed an enlarged para-aortic lymph node measuring 3.1 cm and distal paraesophageal lymphadenopathy measuring 1.7 cm on the largest dimension (Figure 3).

**Figure 4:**
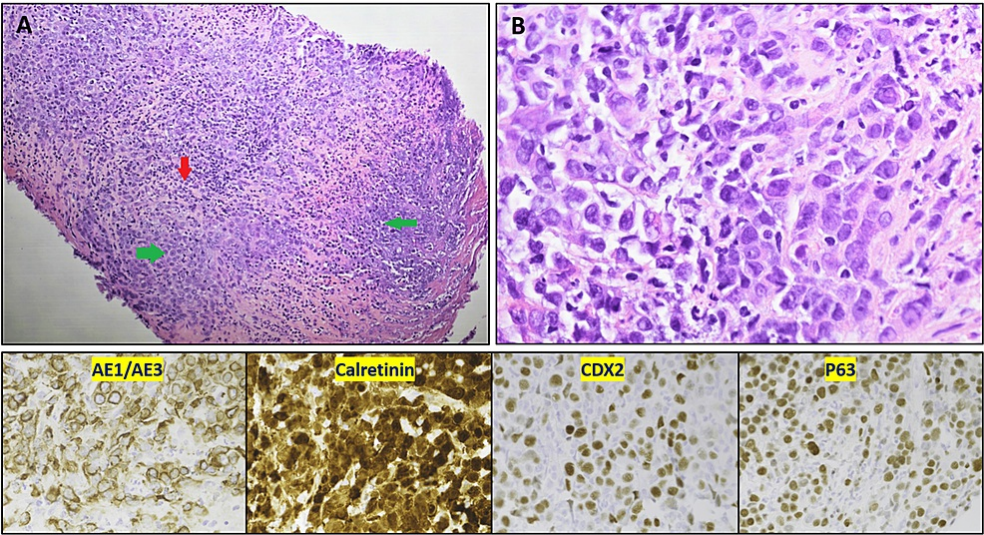
H&E stain of the biopsy at low magnification reveals sheets of poorly differentiated cells (green arrow) in a background of necrosis with abundant intratumoral lymphocytes (red arrow) (A). Higher magnification reveals moderately to severely pleomorphic cells with medium-sized hyperchromatic nuclei, prominent nucleoli, and moderate eosinophilic cytoplasm (B). Immunohistochemically, the tumor cells were positive for CKAE1/AE3, calretinin, CDX2, and p63. H&E: hematoxylin and eosin; CKAE1/AE3: cytokeratin AE1/AE3; CDX2: caudal-type homeobox 2

Given the extensive abdominal lymphadenopathy and concern for a lymphoproliferative disorder, a bone marrow biopsy was done, which ruled out underlying bone marrow disorder. Staging workup using CT chest with contrast showed moderate bilateral pleural effusions. Colonoscopy was inconclusive due to the inability to advance the scope into the cecum because of excessive bowel looping. However, no masses were observed in the large intestine. Carcinoembryonic antigen was within normal limits (0.9 ng/mL). CT-guided biopsy of the right lower quadrant mass showed poorly differentiated malignant neoplasm with infiltrative pleomorphic cells. On immunohistochemistry (IHC), the cells were positive for CKAE1/AE3, GATA binding protein 3 (GATA3), calretinin, p63, and caudal-type homeobox 2 (CDX2) and negative for CK7, CK20, and SATB2 (Figure 4).

**Figure 5:**
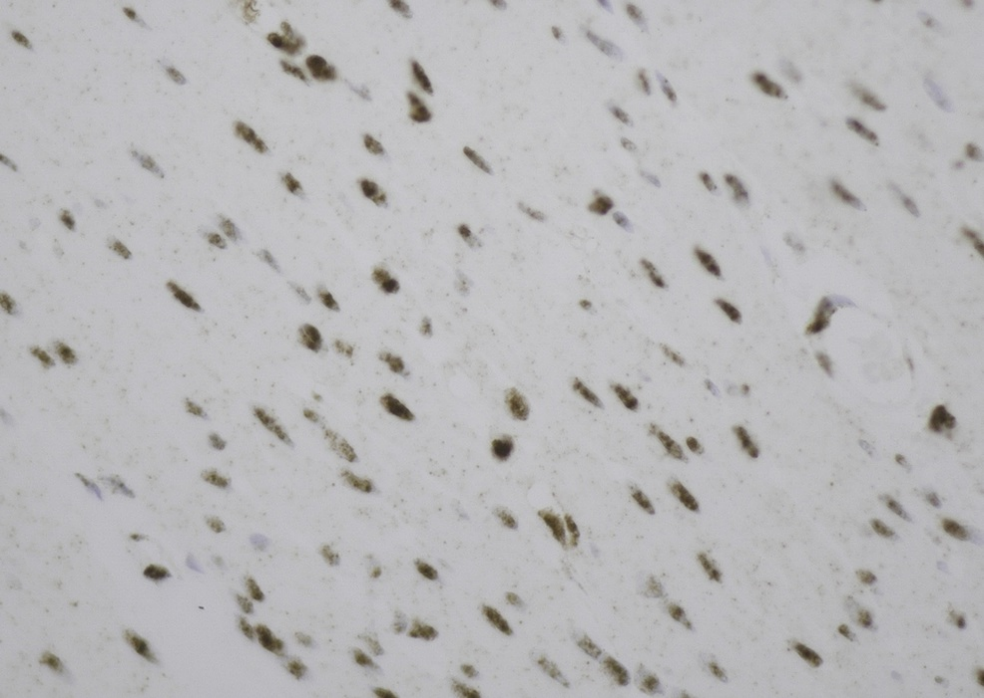
Immunohistochemistry showing loss of PMS2.

Given GATA3 positivity, estrogen receptor and mammaglobin were negative, ruling out breast primary. Wilms tumor gene protein 1 was negative, ruling out mesothelioma. Other stains, including paired box gene 8, Sry-related HMg-Box gene 10, and thyroid transcription factor 1/Napsin A were negative, ruling out ovarian and lung primary malignancies. Further pathology consultation was sought, which reported a poorly differentiated neoplasm with abundant lymphocytic and neutrophilic infiltration along with loss of PMS2 expression with intact MSH6 expression (Figure 5).

A diagnosis of MC of the colon was rendered given a clinical presentation.

## Discussion

MC of the colon is a rare histologic subtype of colorectal malignancy. Only 5-8/10,000 colon cancers have the characteristic histological features of MC [4]. It is slightly more common among middle-aged women and usually presents in the proximal colon than the distal, with rare metastasis to lymph nodes. In a prospective study of 11 patients with MC, most patients had signs of a lower gastrointestinal malignancy, such as melena, abdominal pain, diarrhea, or altered bowel habits, with only one patient developing an abdominal mass [5]. Our patient also presented with nonspecific symptoms, including abdominal pain and weight loss. She also had involvement of the right side of the colon, as seen in most cases [2].

MC was previously called “large cell minimally differentiated carcinoma” as it is morphologically similar to undifferentiated carcinoma. Tumor cells in MC are highly proliferative with abundant cytoplasm and prominent nucleoli and can invade surrounding structures. The World Health Organization further defines that cells in MC are arranged in sheets and lack the glandular formation of colonic adenocarcinoma [6,7]. Definitive diagnostic criteria are lacking, and the various histopathological and immunohistochemical diagnostic criteria vary depending on experience. In our case, histology was nonspecific, showing poorly differentiated malignant cells, thus increasing the diagnostic challenge.

Histology alone cannot always differentiate MC from poorly differentiated adenocarcinoma as both tumor cells show a lack of glandular structure. Therefore, IHC staining is crucial in confirming the diagnosis of MC. MC generally has a high level of microsatellite instability (MSI) and loss of mutl homolog1 (MLH1) [3]. It frequently does not express specific stains for intestinal differentiation such as CDX2 and CK20 but can stain positive for calretinin and CK7 [7]. In our case, the pathology showed a poorly differentiated tumor that was negative for CK20 but positive for CDX2, creating a diagnostic dilemma. Calretinin positivity and loss of PMS2 helped in narrowing our differential. According to a study by Lin et al., 89% of cases were positive for cadherin (CDH17) and SATB2, while calretinin was positive in 67% of the cases. Studies have also shown that 60%-80% of MC are associated with deficient mismatch repair protein and a high degree of MSI. The majority of cases in the literature showed loss of both MLH1 and PMS2 [7,8]. Our case had isolated loss of PMS2 expression. Molecular testing for MLH1 methylation was not performed. Isolated loss of PMS2 is uncommon and seen in about 4% of all CRCs. These tumors may also demonstrate more aggressive behavior than other tumors with MSI [9].

Another interesting aspect of our case was the presentation of bulky abdominal lymphadenopathy, which initially favored the diagnosis of lymphoma. Our case was metastatic at presentation, which is uncommon for MC. In a population-based analysis conducted by Thirunavukarasu et al., out of 74 patients diagnosed with MC, only 10% presented with metastatic disease [10]. Among the 11 patients studied by Jessurun et al., none were metastatic at the time of diagnosis, and only one patient had liver metastasis at the time of presentation [5]. Studies have shown that MC has a favorable prognosis than poorly differentiated and undifferentiated adenocarcinoma [11]. Although it is unclear why MC has improved overall survival, one possibility could be due to the low incidence of metastatic disease at presentation. One study found that patients with MC have five-year mortality of 40% compared to 59% in poorly differentiated adenocarcinoma [12]. According to a meta-analysis of 16 studies, MC has a lower incidence of lymph node involvement than poorly or undifferentiated adenocarcinoma [1]. MC is treated similarly to colon adenocarcinoma. Surgical resection is used to treat localized disease, while chemotherapy is used to treat advanced colon cancer. In a recent phase three study of pembrolizumab versus chemotherapy as first-line therapy for MSI-high advanced CRC, the pembrolizumab group had better progression-free survival (16.5 versus 8.2 months) and fewer treatment-related adverse events [13]. Recent literature has shown that compared with other microsatellite-stable and microsatellite-unstable CRC, tumor cells in MC have a higher number of CD8+ cytotoxic T cells and upregulation of programmed cell death protein 1 (PD-1) and interferon-gamma genes. Upregulation of PD-1 can have potential therapeutic benefits in the future with anti-PD-1 therapy [14]. Our patient, unfortunately, was not tested for PD-1. Knox et al. conducted a retrospective analysis of 102 patients with MC and found that *BRAFV600E* mutation was more common in MC (86%), and these patients were at high risk for Lynch syndrome [11]. Our patient had poor performance status with the Eastern Cooperative Oncology Group of 2-3. The patient and family eventually opted for the best supportive care and hospice. Further molecular testing including next-generation sequencing was not performed. However, based on the KEYNOTE-177 trial mentioned above, the patient would have been a candidate for anti-PD-1 therapy with pembrolizumab. Further studies are needed to investigate different treatment approaches and disease response.

## Conclusions

In conclusion, MC of the colon differs from the adenocarcinoma in terms of clinical and immunohistochemical characteristics. Due to its atypical histopathological presentation, immunohistochemical staining and morphological diagnosis are needed for accurate diagnosis. In addition, it is very commonly associated with high MSI. MC has a better prognosis than poorly differentiated adenocarcinoma. Further studies are needed to elucidate the molecular analysis and treatment regimen for MC.
